# The Historical Development of Immunoendocrine Concepts of Psychiatric Disorders and Their Therapy

**DOI:** 10.3390/ijms161226136

**Published:** 2015-12-04

**Authors:** Holger Steinberg, Kenneth C. Kirkby, Hubertus Himmerich

**Affiliations:** 1 Archives for the History of Psychiatry in Leipzig, Department of Psychiatry, University of Leipzig, Leipzig 04103, Germany; 2 Department of Mental Health, University of Tasmania, Hobart TAS 7005, Australia; ken.kirkby@utas.edu.au; 3 Department of Psychiatry, University of Leipzig, Leipzig 04103, Germany; 4 Department of Psychological Medicine, King’s College London, London SE5 8AF, UK; hubertus.himmerich@kcl.ac.uk

**Keywords:** immune system, cytokines, hormones, depression, schizophrenia, history of psychiatry

## Abstract

Relationships between the central nervous, immune and endocrine systems are a focus of psychiatric research, particularly in depression and schizophrenia. The field has long antecedents. Observed phenomena attributable to these relationships date back to the Neolithic era. Immunoendocrine theories in the broadest sense are recorded in antiquity. In the 19th century, Kraepelin and Wagner-Jauregg reported pioneering clinical observations in psychiatric patients. Von Basedow, Addison and Cushing described psychiatric symptoms in patients suffering from endocrine diseases. The 20th century opened with the identification of hormones, the first, adrenaline, chemically isolated independently by Aldrich und Takamine in 1901. Berson and Yalow developed the radioimmunoassay (RIA) technique in 1959 making it possible to measure levels of hormones and cytokines. These developments have enabled great strides in psychoimmunoendocrinology. Contemporary research is investigating diagnostic and therapeutic applications of these concepts, for example by identifying biomarkers within the endocrine and immune systems and by synthesizing and testing drugs that modulate these systems and show antidepressant or antipsychotic properties.

## 1. Introduction

The three major communication systems in the human body, the nervous, immune and endocrine systems, interact in various ways. The pathways concerned are subject to considerable attention in biological psychiatry research [1,2]. It is both timely and informative to consider the historical development of this inter-systemic approach, including addressing some important epistemological and ethical issues related to the manipulation of hormones. In order to cover the main historical themes in a single article, we will concentrate on two major mental disorders in this article, schizophrenia and depression. Other mental illnesses such as alcohol dependence, attention deficit hyperactivity disorder (ADHD), eating disorders or the variety of phenomena connected with reproduction and sexuality are only mentioned in passing, where they are relevant for a general understanding of immunoendocrine concepts of illness or therapy in psychiatry.

## 2. The Development of Endocrine Concepts of Psychiatric Disorders

### 2.1. Historical Preliminary Remarks

Phenomena attributable to interactions between the endocrine and nervous systems were known in Neolithic times and first documented in ancient times. The underlying physiology, leading in turn to an understanding of neuroendocrine pathways, was not identified until the early 20th century. Initially, endocrine conditions were noted to be commonly accompanied by psychopathological phenomena [2]. Much later it became apparent that typical mental disorders like depression could also occur in endocrine diseases or could lead to secondary endocrine conditions. In this article we will highlight major developments in this conceptual history, from the first description of neuroendocrine phenomena in ancient times through to hormone-based concepts of illness and therapy relevant in psychiatry today.

### 2.2. The Use of Castration to Modify Behavior in Animals and Human Being

In Neolithic times, early humans changed their way of life from hunting-gathering to settled farming, domesticating both animals and plants in the north of the Arabian peninsula. They observed that following castration, *i.e.*, surgical removal of the gonads, male animals became infertile and at the same time grew bigger, developing a higher amount of fat [3]. Further, castration allowed the power of bulls to be employed for human benefit, since only castrated oxen were sufficiently docile for use in agriculture as draught and work animals. In Middle Europe, records of oxen pulling plows date back to the mid-6th century BC [4,5], indicating that castration was then an established practice. As we know today, castration shuts off testosterone, the hormone that makes bulls aggressive [2].

Castration has also been performed in human beings for millennia, e.g., in slaves, eunuchs, those defeated in war, as a medical procedure, and also as a ritual or religious act. Self-castration on religious grounds is referred to in the Bible in the Gospel of Matthew 19, 12. Here Jesus of Nazareth (4 BC–30 AC) tells his disciples: “For there are some eunuchs, which were so born from their mother’s womb; and there are some eunuchs, which were made eunuchs of men; and there be eunuchs, which have made themselves eunuchs for the kingdom of heaven’s sake. He that is able to receive it, let him receive it” [6].

**Figure 1:**
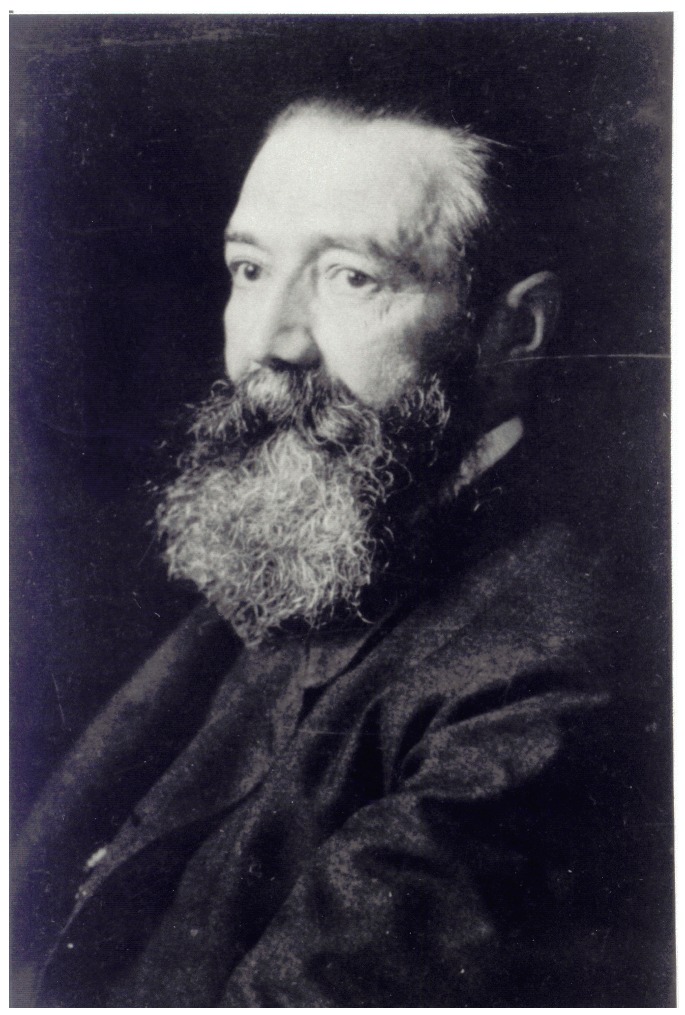
German neuropsychiatrist Paul Julius Möbius (1853–1907) made several contributions to endocrine processes underlying both human behavior and certain illnesses. (Reproduced with permission from: Archives for the History of Psychiatry in Leipzig).

In 1903, the Leipzig-based neuropsychiatrist Paul Julius Möbius (1853–1907) [7,8,9] published a 100-page treatise detailing the effects of castration on the genitals, breasts, bones, body fat, skin, muscles, glands, the inner organs, larynx, skull, the brain and “mental activities”. He also considered aspects of the cultural history of castration [10]. Reflecting on the medical practices of his day, Möbius observed that whilst busy surgeons carried out hundreds of castration procedures in women (removal of the ovaries; oophorectomy), men were seldom castrated. It is well-established that Möbius strongly opposed oophorectomy to treat and allegedly cure women suffering from hysteria. Indeed at the end of the 19th century, removal of the ovaries was a frequent and approved method of treatment for hysteria. The extension of this surgical intervention to illnesses of the nervous system traces mainly back to the gynaecologist Alfred Hegar (1830–1902) [11]. Oophorectomy as proposed by gynaecologists met with divided responses among psychiatrists. It was propounded by Richard von Krafft-Ebing (1840–1902) and Paul Flechsig (1847–1929) as the ultimate intervention in trying to help hysterics. Later the concept of functional neurological disorders found a much greater acceptance [11]. With support from the views of Emil Kraepelin (1856–1926), Möbius’s classification [12] and in particular Möbius’s view that hysteria was mainly a psychological problem became more widely accepted. Over the same period, surgical interventions proved largely unsuccessful, so that finally oophorectomy became disapproved of and conservative measures, above all psychotherapeutic interventions, were developed in place of surgery (Figure 1).

By means of his monograph on castration, Möbius aimed to reduce stigmatization of people who were castrated. It was a generally held view that castrated people were cowards, malicious, curious, fanatical with a great deal of cruelty and greed, deceitful and conceited. Möbius sought to scientifically analyze what impact castration had on the body and character. At a time when hormones had just been discovered and views on them were subject to much controversy, Möbius concluded that the physical and character changes following castration were caused by a “chemical impact” of “certain substances that are released by the glands into the body and which then either inhibit, enhance or change the functioning of the individual organs” [10]. Clearly, Möbius also saw the brain affected by this “internal secretion”, for he maintained that men castrated at a very early age developed lower mental capability, while men castrated at an older age showed an above-average percentage of mental disorders or increased severity of exacerbations (e.g., acute states of excitation combined with hallucinations or longer periods of low mood). By contrast, he maintained, little was known about changes in mental condition in women following castration, other than that women castrated at an older age did not show any marked changes in their mental state. While several cases of mental disorders as a consequence of oophorectomy had been reported in literature, the usual result of castration is the same as the menopause, namely surges of emotion, an increase in excitation and nervousness. Möbius assumed that the latter were based on a “higher stability” of the “female type”, implicating the female brain and its insensitivity to the impact of secretion [10].

Psychiatry too made use of the behavior-modifying effects of castration up until the last third of the 20th century. In the 19th century castration served as a therapeutic option and sedative in cases of uncontrolled rage, epilepsy, hysteria and an inclination to physical violence [11,13]. At the end of the 19th century, Harry C. Sharp in Indiana had 180 male prison inmates castrated to reduce their sexual drive in order to, on the one hand, but not only, prevent sex offences, but also, on the other to prevent masturbation and reproduction of “mental defectives” [14,15]. Degeneration theory and social Darwinism, which emerged in the second half of the 19th century, gave rise to the idea of eradicating unwanted genetic predispositions to mental illness and deviance by general prophylactic castration. The concept of deviance included the notion that antisocial behavior and turning into a criminal both had genetic roots. The idea of the “born criminal” was mainly developed and brought into science by the forensic pathologist and criminologist Cesare Lombroso (1835–1909) from Turin, Italy. It was in 1876 that he first presented his L’Uomo delinquente, one of the foundations of the internationally acclaimed Italian criminal anthropology [16,17]. Castration was applied in forensic psychiatry both in individuals to cure and to prevent relapse. It was also used as a prophylactic to mitigate sexually deviant behaviors. This included homosexuals who were considered as mentally ill based on a genetic predisposition. Nosologically speaking, homosexuals were grouped among psychopaths, or in other words as morally insane or degenerated. Castration in homosexuals reached its peaks during Nazi fascism in Germany when at least several tens of thousands of homosexuals were castrated. Conjecture continues as to the number of homosexuals taken to prison, murdered, mutilated and injured life-long, e.g., by “artificial sexual glands” implanted into their body or through sexual “therapy”. In autumn 1944 the German psychiatrist Nikolaus Jensch (1913–1964) published the then largest and most comprehensive account of “castrated sex offenders”. Jensch concluded that therapeutic outcomes of castration were disappointing, however he did not draw any conclusions regarding clinical practice from that finding [18,19]. Indeed no revisionary conclusions appear to have been drawn elsewhere either, since even after World War II castration of homosexuals was promoted and carried out, with questionable standards of consent. A well-known proponent of this therapeutic approach for many years was the leading German forensic psychiatrist Albrecht Langelüddeke (1889–1977). It was not until the 1970s that castration therapy was replaced by anti-androgen treatment, and the clinical indications restricted to treatment of hypersexuality and pedophilia.

Around 1940, psychiatrist Rudolf Lemke (1906–1957) from Jena (in East Germany) acquired some fame with his hypothesis that homosexuality was caused by hormonal disorders in the diencephalon or the pituitary gland and was hence not a true psychopathic condition, but “merely” accompanied by “psychopathic defects” [20]. The theory that homosexuality was based on disorders in the endocrine system gave rise to hopes of a clear diagnostic criterion, a laboratory test, to diagnose and identify this “sexual pathological deficiency” and in time any sexual deviancy [20]. It should be noted though that 20 years before Lemke, Berlin sexologist and psychotherapist Albert Moll (1862–1939) had rejected hormonal explanations of homosexuality on the basis of the diversity of and great differences in homosexual feelings, lifestyle and behavior. It was for that reason that he also stood up against “gonad experiments” as carried out by physiologist Eugen Steinach (1861–1944) at the Vienna Research Institute for Biology. Steinach had suggested transplanting “heterosexual gonads” into homosexuals and hence some “kind of gonad trade”, which Moll fiercely opposed. This does not mean to say that Moll raised doubts as to homosexuality being an illness. For him it was an illness, yet one that could not be treated somatically or with the help of drugs, but only through psychotherapy [21].

For the authors of the present study, the use of castration as an endocrine therapy in human beings, the more so for a dubious indication, is one of the darkest and hitherto least-researched aberrations in the history of psychiatry.

### 2.3. Humorism and Mental Illness

Based on the theory of the four elements developed by Empedocles in the 5th century BC, Hippocrates (460–370 BC), the renowned doctor of antiquity, elaborated the so-called humoral theory. The human body is held to be filled with four basic substances or bodily fluids, called humors, namely blood, yellow bile, black bile and phlegm. If they are in balance, the person is healthy and this state of body (and mind) was called “eucrasia”. Any deficiency or excess of one or more of the fluids was considered the cause for the emergence of illnesses and referred to as “dyscrasia” [18]. Melancholia (Greek μελαγχολια) for example was defined by Galen of Pergamon (129–216 AD), who summarized the medical knowledge of his time and stood in the tradition of the humoral theory, as an excess of black bile, hence the name (μελαζ—black; χσλη—bile). Humorism was to determine medical thinking for almost 2000 years, having an impact as recently as the 19th century. This despite the fact that long before the 19th century facts were revealed which shook the fundaments of the theory of the four bodily fluids. So when the English researcher William Harvey (1578–1657) described the basics of blood circulation, this made it obvious that the theories of how the blood was composed, which were taught until the end of the Renaissance period, were no longer up to date [22].

In 1602 Felix Platter (1536–1614), the municipal doctor of Basel in Switzerland and professor at the local university, who still followed humoral theories [23,24], described a psychosis occuring during pregnancy, which was the first description of postpartum psychosis in the German-speaking medical literature. In his book “Observationes” of 1614, which contains several case descriptions of mental illnesses, he provided the following characterization: “Right after becoming pregnant, the landlord’s wife was seized by (…) the reprehensible desire to kill the baby she had carried in her body right after giving birth and breastfeeding him or her. She was tormented by this obsessive idea for several months before coming to herself again. (…) On several occasions she thought of killing herself. (…) One day she came to my house and tearfully set out her case. Later, on her way out, she would have drowned herself in a tub full of spring water that stood in the middle of the vestibule, had I not jumped forward and held her back. Eventually, the madness that had seized her during pregnancy was cured by bloodletting, purgations and repeated administration of emetics” (quoted from [24], translation by the authors).

### 2.4. Psychopathological Symptoms in Circumscribed Endocrine Disorders

**Figure 2:**
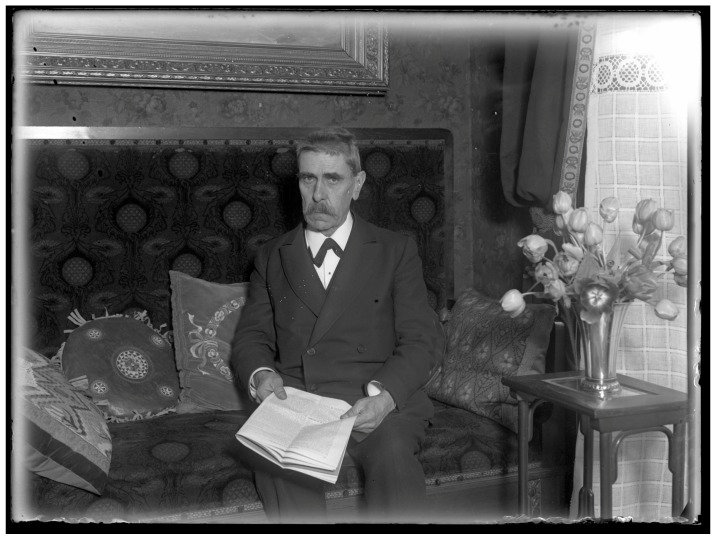
The Austrian neuropsychiatrist Julius Wagner-Jauregg (1857–1940) is the most outstanding researcher in psychiatric immunology. His observation that a vaccination with malaria produced a relief in symptoms or even cure in progressive paralysis was a first landmark in psychiatric therapy by influencing the immune system (Reproduced with kind permission from: Österreichische Nationalbibliothek/Austrian National Library, ID 66321–B).

It is now a well-established fact that without treatment persons with endocrine disorders of the thyroid, adrenal and pituitary glands such as cretinism, Hashimoto’s thyroiditis, Grave’s disease (Morbus Basedow), Addison’s disease, Cushing’s syndrome and pheochromocytoma, may develop severe psychopathological symptoms. Hence at a time when the endocrine causes of these diseases were not yet established, at the turn of the 19th into the 20th century, general practitioners, psychiatrists and neurologists, and surgeons, who dealt with these conditions, uncovered major pathophysiological connections. Vienna psychiatrist and neurologist Julius Wagner-Jauregg (1857–1940) for example carried out fundamental research on hypothyroidism. Based on his studies on cretinism, he dealt with the problem of goitre, treating cretinism with thyroid extract and countering goitre with minor doses of iodine [25]. As will be detailed below, Julius Wagner-Jauregg also played an essential role in developing immunological concepts of illness in psychiatry (Figure 2).

In 1883 the Swiss surgeon Theodor Kocher (1841–1917), who was awarded the Nobel Prize in 1909 for his works on the physiology, pathology and surgery of the thyroid, described the effects of hypothyroidism on behavior in his study “On goitre extirpation and its consequences”. For his study, Kocher had compared patients who had had their thyroid partly resected and patients who had had their thyroid fully resected and came to the conclusion that: “Those who had had a partly excision done were (…) all healthy. Patients who had undergone total excision however show (…) considerable inhibitions of general well-being. Soon after being released from hospital, the patients complained of being tired (…), feeling cold and of their mental energy and capability declining. (…) Apart from their thinking being slow, gradually they also talk and move much more slowly. If we were to give the illness described a name, we would need to choose one reflecting the similarity of the symptoms with those of idiotism and cretinism” [26].

Paul Julius Möbius, who has been mentioned earlier, is another example, a neuropathologist who researched the connections between thyroid diseases and psychopathological conditions. It was he who had—for German-speaking psychiatry at least—established the correct etiological cause for Grave’s Disease (Morbus Basedow). Before him, in the 19th century the condition had been classified as a neurosis without an anatomic basis [27] or as a nervous disease somehow caused by the cervical sympathetic trunk. It was in 1840 that general practitioner Carl Adolph von Basedow (1799–1854) from Merseburg described the later so-called “Merseburg trinity” of exophthalmus, goitre and tachycardia [28]. In 1886/87 Möbius concluded, most likely for the first time ever, that a “noticeable change in the thyroid was the most probable cause” for Grave’s disease [29]. He had come to this conclusion by recognising that the opposite of Grave’s disease was myxedema: “Here the thyroid is enlarged, there the thyroid is reduced in size, here an acceleration, there a slow-down of the heart’s functioning, here a general reduction and there an increase of the skin’s thickness, here the skin is hotter, there colder, here mental agitation, there apathy” [30]. In particular Möbius assumed that the thyroid had a poisonous impact on the organism, which was the reason for Grave’s disease to emerge. Having established the course, Möbius started to search for an antidote to the poison released by the thyroid and developed “Möbius’s anti-thyreoidin”, which however failed to produce a lasting positive effect. Yet what is still true is that his drug, which Möbius and the Darmstadt-based pharmaceutical company Merck derived from the blood of wethers (castrated rams) whose thyroid glands had been removed, was one of the first glandular pharmaceuticals [8]. A therapeutic option with a lasting effect on Grave’s disease, namely radioiodine therapy, was not developed before 1942 by Saul Hertz (1905–1950) and Arthur Roberts (1912–2004) [31]. One year later, Edwin B. Astwood (1909–1976) presented an alternative therapy with his antithyroid agent Thiouracil [32].

As far as the adrenal gland is concerned, it was the English doctor Thomas Addison (1793–1860) who pointed the way towards our modern understanding of it in 1855. In that year he first described the disease that still carries his name. Addison’s disease is a syndrome caused by destruction of the adrenal gland through tumors, tuberculosis or auto-immune disorders. Characteristic symptoms observed and described by Addison included tiredness and general weakness, indigestion, cardiac rhythm and pigmentation disorders [33]. In 1912 surgeon and neurologist Harvey Williams Cushing (1869–1939) described the “polyglandular syndrome“, which is considered the first description ever of Cushing’s syndrome. Twenty years later, in 1932, Cushing added the description of a basophilic adenoma within the adenohypophysis, in patients with Cushing’s syndrome showing the typical symptoms of moon face, plethora, translucent stretch marks (striae rubrae) and truncal obesity [34,35]. Moreover Cushing found out that patients suffering from this endocrinological disorder developed symptoms of anxiety and depression [36].

### 2.5. The Discovery of the Hormones

Early 20th-century experimental medicine, which was based on scientific principles, laid the foundations for uncovering the endocrine, biochemical and neurophysiologic circuits. In 1901 Thomas Bell Aldrich (1861–1938) and Jokichi Takamine (1854–1922), independently from each other, succeeded in isolating adrenalin [37,38]. This was the first hormone to be described in its pure form and structure. One year later, in 1902, William Bayliss (1860–1924) and Ernest Starling (1866–1927) found out that, when stimulated with hypochloric acid, the intestinal wall released an agent that stimulated secretion in the pancreas. They called this agent “secretin” [39]. In 1905 Starling proposed “hormone” as the generic term for any messengers that are released by endocrine glands into the blood to stimulate the activity of organs, such as secretin. The first decades of the 20th century brought the discovery of thyroxine and cortisone by Edward Calvin Kendall (1886–1972) [40], insulin (1921) by Frederick Grant Banting (1891–1941), Charles Best (1899–1978) and Nicolae Paulescu (1869–1931) [41] and of the sexual hormones–mainly by Adolf Butenandt (1903–1995). In 1931 Butenandt isolated androsterone in its pure form and four years later he succeeded in synthesizing and establishing the structure of testosterone [42]. In 1939, Butenandt, who at that time was head of the Kaiser-Wilhelm (later Max-Planck) Institute for Biochemistry in Berlin, was awarded the Nobel Prize for Chemistry for his works on the steroid hormones. In 1950, Kendall, Tadeus Reichstein and Philip S. Hench were awarded the Nobel Prize for Medicine for uncovering and analyzing the hormones of the adrenal cortex.

From the point of psychiatry, all relevant hormones had been discovered and described: namely the glucocorticoids, the thyroid hormones and the sexual hormones. It was not before the end of the 20th century that appetite regulating leptin and ghrelin, which are possibly pathophysiologically essential for eating disorders, were discovered [43,44].

### 2.6. The Emergence and Systematic Development of Endocrine Concepts of Illness in Psychiatry

In 1908 the French psychiatrist Paul-Marie Maxime Laignel-Lavastine (1875–1953) published several studies on the connection of internal secretion and mental illnesses such as psychoses and melancholia and called this new field of study “psychiatrie endocrinienne” [45]. In the 1940s the Swiss psychiatrist Manfred Bleuler (1903–1994) began to systematically analyze endocrine illnesses and mood changes resulting from them. He found out that on the one hand psychoses emerged much more frequently at times of endocrine rearrangements such as giving birth or menopause and that on the other endocrine illnesses were often accompanied by mood disorders, changes in sensitivity towards their own body (cenesthesia) and in libido. On the basis of these findings, Bleuler concluded that “mental and endocrine circuits interact and augment each other to a high degree” [46]. Moreover, his 1954 monograph titled “Endocrinological Psychiatry” is the first comprehensive overview of the different hormonal circuits, their disorders plus their impact on and consequences for the emergence of mental illnesses. On the other hand, he also summarized distinctive endocrinological changes found in patients suffering primarily from mental disorders, such as affective disorders or schizophrenia [2,47]. Even now, more than 50 years after the publication of this book, Bleuler’s methodological approach and his combining of endocrinological and neurophysiologic methods can still be found in current psychiatric research, e.g., in [48]. In 1979 Bleuler defined endocrinological psychiatry as “the study of mental changes in endocrine illnesses and of endocrine changes in mental illnesses”. Endocrinological psychiatry included “the study of endocrinological treatments in psychiatry and of psychotherapeutic therapies in endocrinological disorders” [49].

In women, during periods of changes of hormone levels the risk of developing an affective or psychotic disorder is significantly increased. Such periods of large variations in hormone levels are puberty, the premenstrual phase, pregnancy, the post-natal period and the menopause [50]. The first scientific discussions of postpartum psychosis in German-speaking psychiatry are: the report by Platter [24] mentioned above; the accounts by Johann Georg Schenck von Grafenberg (1560–1620) of two cases of puerperal mania in his “Observationum medicarum, rarum, novarum, admirabilum et monstrosarum” (1609); the MD doctorate theses by Gottfried Schultzius in Frankfurt/Oder (1705), Johann Justus de Berger in Göttingen (1744) and Wolfgang Thomas Rau in Nuremberg (1752) [23]. One of the earliest more comprehensive monographs on the topic was published in 1877 by Ludwig Hugo Ripping (1837–1898) and titled “Mental disorders in expectant mothers, women in childbed and breastfeeding women”. This book contains a brief historical introduction and references to the literature on the topic published before that date [51]. The fact that there are several doctorate theses in the 19th century that deal with aspects of post-natal mental disorders is based on the high percentage of women suffering from such conditions among patients newly admitted to psychiatric asylums. In the Siegburg asylum, where Ripping worked, 22% of the newly admitted patients in 1877 were women suffering from puerperal psychosis. Martin Schmidt, who worked as a doctor in Liegnitz (present-day Legnica in Poland, then in the German province of Silesia) and carried out a comprehensive analysis of patient admissions to the Silesian Provincial Asylum of Leubus (today Lubiąż), found that 17.3% of the women admitted suffered from childbed psychoses [52]. It should be noted that about half of the patients suffering from these conditions, which were then referred to as “madness, idiocy, circular insanity” and corresponded to the present-day diagnoses of schizophrenic or schizophreniform disorder, or puerperal psychosis, could not be helped in pre-psychopharmaceutic times. In Schmidt’s survey, the percentage of these women was as high as 80%. Today the prognosis with psychopharmaceutic treatment is much better, since virtually all patients can reach remission. However, the probability of the illness recurring in a later pregnancy is still as high as 50% [53]. The first reports on mental disorders connected with menstruation date back to the early 19th century. In 1870 Carl Emil Louis Mayer (1829–1890) published a 160-page monograph on the effect of changes in female sexual organs on mental health titled “Die Beziehungen der krankhaften Zustände in den Sexual-Organen des Weibes zu Geistesstörungen” (“The connections of pathological conditions in female sexual organs with mental disorders”). This book also covered menstruation-based mental disorders [54]. The chapter on sexual hormones in the above-mentioned book on “Endocrinological Psychiatry” by Manfred Bleuler of 1954 features a comprehensive summary of basic findings on the connections between hormonal phases of the menstrual cycle and mental illness in women [47].

### 2.7. Therapeutic Use of Various Hormones in Psychiatry

In the 1910s and 1920s the first drugs were released and marketed for their hormone-based agents and effects. They were also soon used in psychiatry. In 1914 Iwan Bloch (1872–1922) launched two pharmaceuticals called Testogan and Thelygan. In addition to yohimbine they were advertised to contain “extracts of the male and female gonads” and to be allegedly effective for not only “sexual dyshormonia and insufficiency”, premature aging, metabolic disorders or “heart neuroses”, but also in neurasthenia, then a commonly applied diagnosis, and depressions. The compound preparation of the two agents proved a commercial success for its producer [55]. After insulin had been discovered in 1921 [41], it was also soon applied in psychiatric patients, on the one hand to end refusal to eat and on the other to treat delirium tremens. The Austrian-Jewish psychiatrist Manfred Sakel (1901–1957) found that in a patient suffering from diabetes who was addicted to morphine the addictive behavior and urge declined during a phase of hypoglycemia. In 1921, he started to test the effects of insulin shock in all of his patients. After he was expelled from Berlin in 1933, he continued his studies in Vienna and found hypoglycemic shock to be particularly effective in schizophrenic patients, when insulin was applied in large doses over a longer period of time to put the patient into a comatose state [56]. Under the names insulin coma therapy (ICT) or insulin shock therapy (IST) modified versions of the new treatment option became a standard treatment, besides electro(convulsive)shock (ECS) and cardiazol compulsive shock therapy, for schizophrenia and depression, especially after Sakel emigrated to the USA in 1936 and started to widely apply it there. Yet it should be noted that Sakel was never able to provide a valid explanation as to why and how his therapy produced its effects. What is more important is that it was widely used until the 1960s despite its possible severe side effects, including the death of patients, despite being cost-intensive and despite the fact that it only helped about one third of patients. Due to its high costs and complicated use, over time Sakel’s therapeutic approach was pushed into the background and finally replaced by more modern psychopharmaceutic drugs [57].

Other hormones tried on patients suffering from schizophrenia or affective disorders included chorionic gonadotropin, testosterone and oxytocin, but these did not become valid therapeutic options [58,59]. After cyproterone acetate showed antiandrogenic effects in rats in 1966, it was also tested in men with a pathologically increased sex drive or disturbed sexual preferences [60]. This agent has held a valid indication for use in hypersexuality and sexual deviancy until the present day. Currently luteinizing hormone releasing hormone (LHRH) antagonists, including leuprorelin acetate in particular, are tested as alternative therapeutic options [61].

### 2.8. Technical Developments for Establishing Hormone Concentration in the Blood

In the 1960s new technologies to measure hormones such as the radioimmunoassay (RIA) were developed and several chromatographic, analytic and synthetic methods refined. Together with progress achieved in molecular biology, this gave a new prominence to neuroendocrinology. RIA was first described and introduced into medical diagnostics in 1959 by Solomon Aaron Berson (1918–1972) and Rosalyn Yalow (born 1921) [62]. The first protein measured for diagnostic purposes with the help of RIA was insulin. For developing this new diagnostic method, Rosalyn Yalow was awarded the Nobel Prize for Medicine in 1977. With the help of RIA and the later developed enzyme-linked immunosorbent assay (ELISA) it became possible to measure nano- and picomolecular concentrations of hormones in the blood.

## 3. The Development of Immunological Concepts of Psychiatric Disorders

### 3.1. Observations and Hypotheses in the Ancient World

Observations on a possible connection of brain illnesses with an activation of the immune system date back to ancient times. Hippocrates found that some epileptics who became infected with malaria were cured from their epileptic illness. He further stated that “Those who were taken by quartan fever do not fall prey to the great disease (epilepsy) and in those who had been suffering from it the illness stops once quartan fever comes in” (quoted from [63]; translation into English by the authors). Galen confirmed this connection and called this transition from one illness into another “metaptosis”. Galen reported other instances of metaptosis or the positive effect of fever on illnesses, e.g., on lethargy [64]. However we wish to make clear that saying that Galen understood the positive effect of fever on epilepsy or lethargy as the consequence of the activation of the immune system would mean reading present-day knowledge into his observation, which has nothing to do with the conceptualization of illnesses at the time. Still, according to Hippocrates and his students, fever, in particular acute fever, was a key natural form of healing. Fever was understood as an increase in “vital warmth”, *i.e.*, body temperature, which led to “boiling up” an imbalance of the four bodily fluids, so that it could be eliminated through the intestine, urine or perspiration [60]. These humoral concepts, whose basics are described above, remained valid for many centuries. It was not until the 17th century that researchers challenged this view and found a different approach and improved understanding of the human body by measuring its various parameters.

### 3.2. Measuring Body Temperature and Blood Analysis

In 1674 Antoni van Leeuwenhoek (1632–1723), a researcher from Delft in the Netherlands, first described the red blood cells that had first been seen by his fellow countryman Jan Swammerdam (1637–1680) under a microscope in 1658. This laid the basis for understanding blood not as a uniform juice, but a compound of different components. The first measurement of fever with a thermometer is ascribed to Santorio Santorio (1561–1636) in Padua, Italy, in 1626.

Chemist and doctor Georg Ernst Stahl (1659–1734) from Halle, Germany, was one of the fathers of the so-called animism theory that took the soul (anima) as the decisive starting point of everything that happened in the body, *i.e.*, of all chemical and physical processes in the body. In 1700 he described both fever and inflammations as natural healing powers or as defensive reactions of the body to infections. According to Stahl, the soul built up and strengthened the body and protected it by helping it to fight illnesses, mainly by activating circulation in the body to eliminate the bad juices (see humorism). Through the activation of circulation, more good, clean blood would pass through the affected organ and cleanse it. By hypothesizing active hyperaemia controlled by an increase in fibre tone, Stahl anticipated the theory of inflammations by John Hunter (1728–1793) [65].

Standardised measurement of body temperature became possible after Daniel Gabriel Fahrenheit (1686–1736) invented the mercury thermometer and Anders Celsius (1701–1744) put forward his temperature scale in the 18th century. Yet it was not until the 19th century that Carl Reinhold August Wunderlich (1815–1877), professor of medicine and head of the university hospital in Leipzig, Germany, introduced standardised measurements of patients’ body temperature and scientific empiric clinical research. (for Wunderlich’s influence on the history of psychiatry see [66]). Only then did psychiatrists start investigating correlations of body temperature, which is essentially controlled by messengers of the immune system, and both how their patients behaved, experienced their illness and how their symptoms changed.

It is little known that the prominent German psychiatrist Emil Kraepelin (1856–1926), who was a student of Wunderlich, qualified as a university lecturer in Leipzig with the help of a publication that focused on organic psychiatric disorders following acute inflammatory diseases. His habilitation thesis attempted to sub-classify them into disorders appearing when the fever rises and those occurring when it falls. In fact Kraepelin’s hypotheses on the psychoimmunological mechanisms behind the emergence of psychiatric symptoms and mental disorders following acute infections compare very well with later developments and current psychoimmunological explanations.

In particular, Kraepelin discussed that it is probable that in general fever can cause mental illnesses. He suggests that this might be due to the fact that fever has an impact on both the nerves and their conductivity and on metabolism. As for the latter, fever could lead to an increase in the amount of breakdown products in the central nervous system. This in turn could lead to changes in the blood flow within the central nervous system and in the final outcome to pyemia, septicaemia, uremia or metabolic changes. Kraepelin suggested that these changes are induced by enzymes stemming from infectious agents. In his paper, Kraepelin reviewed the literature available at the time regarding the most important acute diseases reported to be associated with psychiatric disorders and found that it was in particular malaria, measles, smallpox, acute rheumatism and scarlet fever that have shown firm links with mental disorders. Other acute illnesses causing such disorders include pneumonia, pleurisy, erysipelas, typhus and cholera.

The last part of his thesis is a statistical meta-analysis of published studies and case reports with regard to the frequency of psychiatric disorders with respect to the different acute diseases, differentiated by gender, age, individual disposition, duration and outcome of the psychosis. In total, Kraepelin included about 700 cases and analyzed these cases as a whole group and in addition to that in sub-groups such as the groups of “febrile psychoses”, by which he meant psychoses associated with the rise of body temperature, and “asthenic psychoses” by which he understood psychoses that occurred when the body temperature decreased, e.g., when patients recovered from a disease [67,68].

At the first glance, Kraepelin’ habilitation thesis seems to be a mere compilation of contemporary studies on organic or symptomatic mental disorders co-occurring with acute and feverish diseases. Yet Kraepelin went beyond that and drew conclusions regarding the interrelationship of fever and mood as well as fever and appetite regulation, which may be interpreted as early speculations about psychoimmunological mechanisms. These were revived scientifically after cytokines were discovered, such as tumor necrosis factor-α (TNF-α) in 1975 [69].

Significant advances in modern optics by Ernst Abbe (1840–1905), Carl Zeiss (1816–1888) and Otto Schott (1851–1935) on the one hand, and the development of a dyeing method for blood by Artur Pappenheim (1870–1916) together with that of a hemacytometer by Viktor Schilling (1883–1960) on the other created the technical prerequisites to differentiate blood components. Psychiatrists then began investigating for potential specific findings in the blood count of psychiatric patients. One of the first to do so was Scotsman Lewis C. Bruce (1866–1945) at the Perth District Asylum in Murthly. Together with his assistant Alexander S.M. Peebles he published a report on particularities in the blood count of patients suffering from mental illnesses. They found leucocytosis and eosinophilia in “catatonic” patients, under which name they subsumed all patients suffering from what is now referred to as schizophrenia [70]. Originally they presumed that these particularities could have been caused by an infection with streptococci which led them to try infecting rabbits with streptococci extracted from the blood of patients with schizophrenia [71]. Rabbits treated in this way developed behavior that would nowadays be categorised as “sickness behavior”, but not schizophrenic symptoms as Bruce and Peebles had hypothesised. Their observation made the two Scotsmen suggest that vaccinating patients against streptococci could have a positive effect. Following animal experiments they tested vaccination with streptococci antiserum on patients with acute schizophrenia [72]. In 1930 the American hematologist William Dameshek (1900–1969) found a reduction in the count of polymorphonuclear cells, lymphocytosis and eosinophilia in about 30% of patients suffering from dementia praecox, or schizophrenia [73].

Probably the most outstanding researcher in psychiatric immunology was the above-mentioned Julius Wagner von Jauregg [74,75]. In 1927 he was awarded the Nobel Prize for Medicine for his observation that a vaccination with malaria produced a relief in symptoms or cure in progressive paralysis, or neurosyphilis, which from a present-day perspective was a landmark development in psychiatric therapy [76,77,78]. His early and lasting interest in experimental pathology and later work as assistant to Samuel Stricker (1834–1898) at the Vienna Institute of General and Experimental Pathology proved influential for his later work as a psychiatrist. He became assistant doctor to Max Leidesdorf (1819–1889) at the psychiatric clinic of Vienna University in 1883 and six years later, aged 32, professor of psychiatry and head of the psychiatric hospital of Graz University, Austria, succeeding the renowned Richard von Krafft-Ebing. Ten years later he once again took over from Krafft-Ebing when he returned to Vienna as professor and head of the 1st psychiatric clinic of Vienna University, which at the same time functioned as Provincial Asylum of the province of Lower Austria.

As early as during his assistant years Wagner-Jauregg started meticulously collecting case histories of patients who developed a febrile disease or episode observing that in certain mental illnesses the symptoms remitted during or through the fever. He also found cases in which patients with an extremely unfavourable prognosis were cured after they suffered from an intercurrent disease such as thyphoid, cholera, malaria intermittens, acute exanthema or erysipelas [79,80].

What is today understood as psychoimmunological research formed a major aspect of Wagner-Jauregg’s work on psychoses and neurosyphilitic illnesses. He started dealing with this aspect in the 1880s and continued doing so well after being awarded the Nobel Prize for Medicine. It should be noted that Wagner-Jauregg did not pigheadedly follow his prize-winning malaria fever therapy, but also analyzed other approaches. At the age of 82 he published a paper in the Vienna Clinical Weekly asking if chemotherapy or producing higher temperature by physical means could be more effective ways to trigger a curing fever [81]. In 1928 Wagner-Jauregg retired and twelve years later he died, not living to see the invention of the first antibiotic drugs. In the history of psychiatry however his psychoimmunological therapeutic approach is of outstanding importance, marking the end of “therapeutic nihilism”, when psychiatrists could offer little to no help to their patients. On the contrary, his approach is the first therapy addressing the cause (killing the spirochetes infecting the brain in tertiary syphilis) in the history of psychiatry.

### 3.3. The Emergence of Immunology

Traditionally, the emergence of immunology is marked by the first vaccination of a boy with cowpox followed by an infection with smallpox by Edward Jenner (1749–1823) in 1776 and the development of a special vaccine against rabies by Louis Pasteur (1822–1895) in 1885. In 1890 Emil Adolf von Behring (1854–1917) found what he called “anti-toxines” in the serum of patients who had overcome diphtheria. This and other findings helped Paul Ehrlich (1854–1915) to develop the theory of antibodies, which he presented in 1900. After the basis was laid, other cellular and humoral components of the immune system were identified, pathogens found and described and the differences in endogenic and exogenic processes discovered. At the beginning of the 20th century, immunology showed rapid advances.

At about that time Emil Kraepelin and other leading neuroscientists of the time postulated that schizophrenia, then named dementia praecox, was caused by an organic destructive process in the brain [82,83]. In the 1930s, this led Hermann Lehmann-Facius (1899–1960), a neuropathologist at the University of Frankfurt/Main, Germany, to investigate whether this destructive process could be caused by an auto-immune reaction, whereby antibodies against structures in the brain were produced. Lehmann-Facius further presumed that in particular so-called “brain lipoids” were affected, which are biochemically specific substances in the brain tissue. To verify his hypothesis, he produced extracts from unaffected brain tissue containing such lipoids and had them react with both the spinal fluid of unaffected individuals and of patients suffering from schizophrenia (a so-called brain lipoid reaction). In the latter group, 95% showed a flocculation which made Lehmann-Facius suggest that indeed their spinal fluid contained antibodies against the brain lipoids (“Hirnlipoidantikörper”). As a consequence of his studies, he also analyzed the brain lipoid reaction in other mental and neurological illnesses and found that in multiple sclerosis and also brain tumors, flocculation occurred which made him suggest that in these illnesses too, at least in a certain sub-group of the patients, specific brain lipoid antibodies are formed, which are causal for the destruction in the brain [84]. Following these first tests, Lehmann-Facius conducted more experiments with an extract from a patient suffering from catatonic schizophrenia. With this extract he found a positive brain lipoid reaction in 100% of the patients with schizophrenia and postulated a “complete specificity” [85]. Yet soon criticism was raised against his results, in particular by auditors visiting his laboratory. They were especially suspicious about the fact that in each test, the diagnosis of the patient whose spinal fluid was being used was known–and about the fact that flocculation could be elicited “if one shook up the sediments properly” [86]. Even though Lehmann-Facius’s results could not be reproduced due to a lack of reliability, they can still be counted as an early attempt to investigate a suggested auto-immune cause of schizophrenia. (It was not until many years later, when cytokines were discovered and could be measured, that this “auto-immune hypothesis” for schizophrenia became outdated.)

### 3.4. Technical Developments in Measurement of Cytokines and Immunologically Relevant Cells

Essentially important for progress in psychiatric immunology and psychiatric endocrinology were the discovery of RIA and its enhancement into ELISA. These two developments made it possible to establish the concentration of cytokines, *i.e.*, the messengers of the immune system, in the blood. Together with the established clinical and immunological diagnostic methods, *i.e.*, the documentation of the course of certain parameters like body temperature, the sub-differentiation of the blood compounds and the description of immunostimulation paradigms, the basis was laid for our current understanding and model of the immune systems and for modern psychoimmunological concepts of illnesses. Basically, the immune system refers to the biological system of defence in advanced organisms to prevent the organism from being damaged by pathogens through identification of these pathogens as foreign and eliminating them.

**Figure 3:**
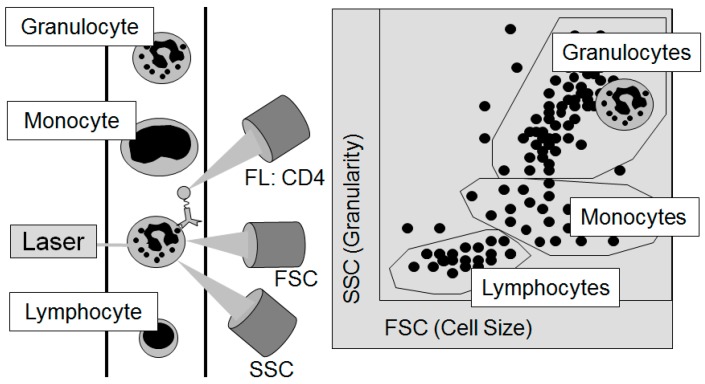
The principle of flow cytometry to differentiate the individual cellular components of the blood: The Forward Scatter (FSC) measures the acute-angular diffraction of the light and depends on the cell’s volume. The Sideward Scatter (SSC) measures the rectangular diffraction of the light and depends on the granularity of the cell, the size and structure of its core, and the amount of vesicles contained in it. With the help of FSC and SSC, the individual cells contained in the blood can be differentiated. For further sub-differentiation, e.g., by the sub-type of cell or its functional status, marker molecules on the surface of the cells, the so-called cluster of differentiation (CD), are used. In this figure, a granulocyte passes the laser light (left part of the figure). After sorting by cell size and granularity according to the light detected by the FSC and the SSC as displayed in the right part of the figure, the granulocyte could then be further analyzed for the frequency of CD4 on its surface by the signal of fluorescent (FL) antibodies against CD4.

A final landmark in the technical advancements of the 20th century and the characterization of the immune system is the development of fluorescence-based impulse cytophotometry, which is today better known under the name of fluorescent-activated cell sorting. This new technology allows further sub-differentiation of the individual cells of the immune system. The method was developed by radiobiologist Wolfgang Göhde (born 1940) at the University of Münster in Westphalia, Germany, in 1968 [87] based on research by US electrical engineer Wallace H. Coulter (1913–1998) and his patent of 1949 for counting particles dissolved in liquids. The working principle behind this technology is that a cell emits optical signals when it passes through a laser beam. For impulse cytophotometric measurements the cells dissolved in a solution are sucked into the sensor module by a capillary and in there they pass the laser beam one by one. The light which is emitted through the passage is recorded by specific detectors, whereby the amount of light scattered correlates with both the size of a cell and its complexity. Due to the huge amount of vesicles contained in them, granulocytes for example emit a significant pattern of light differentiating it from other lymphocytes (Figure 3).

These advances in cell differentiation go back to César Milstein (1927–2002), an Argentinian molecular biologist, who developed the so-called monoclonal antibody technology in Cambridge, UK, in 1975 together with his assistant Georges Jean Franz Köhler (1946–1995), who came to England from Munich, Germany. With the help of monoclonal antibody technology, certain types of myeloma cells are fused with a B cell to produce huge amounts of monoclonal antibodies [88]. For their technology, Milstein and Köhler, together with the Danish immunologist Niels Kaj Jerne (1911–1994) were awarded the Nobel Prize for Physiology or Medicine in 1984. First, Milstein and Köhler succeeded in producing one type of monoclonal antibody, effective against one certain kind of CD, which was hence called CD1. Over the following years many more specific monoclonal antibodies were discovered so that a standardised system of differentiation of these CD was needed. The foundations for the present-day CD classification were laid in 1982 at the 1st International Workshop and Conference on Human Leukocyte Differentiation Antigens (HLDA) in Paris.

Impulse cytophotometry is one method to identify regulatory T cells (so-called Tregs), since Tregs express a lot of CD4 and CD25 and can hence be marked with the help of the above-mentioned specific antibodies, which stick to them and are made to fluoresce in the cytophotometer. The specific fluorescence detectors then identify the amount of the antibodies, which in turn is the measure for the quantity of Tregs in the blood. Regulatory T cells inhibit or suppress the production of pro-inflammatory cytokines and are hence one mechanism of controlling the immune system.

## 4. Current Immunoendocrine Concepts of Psychiatric Disorders and Their Therapy

### 4.1. Current Hormone-Based Concepts of Depression

In the mid-1960s, for the first time significantly increased concentrations of cortisol, a stress hormone, were found in patients in the phase of falling ill with major depression, while over the course of clinical treatment and remission of symptoms its concentration went back to normal after a rather short period of time [89,90]. Since then numerous investigations have studied how neuroendocrinological regulation changes in patients with depression. The results have confirmed that changes in the regulation of the hypothalamus-pituitary-adrenal (HPA) system are one of the most consistent neurobiological features of affective disorders.

In current psychiatric practice, the so-called combined dexamethasone (Dex) suppression and corticotropin-releasing hormone (CRH) test (Dex/CRH test) is frequently administered to aid diagnosis of an affective disorder [91]. As the name suggests, the combined Dex/CRH test consists of two stages. On the first evening, patients take a 1.5 mg dexamethasone pill. At 3 p.m. the following day, blood is taken to establish how much of the cortisol and adrenocorticotropic hormone (ACTH) production is suppressed by the pill. Directly afterwards, at 3:02 p.m., the patient is injected with 100 μg CRH intravenously. Over the course of the hours directly after the injection, repeated blood samples are tested for the amount of cortisol and ACTH released into the blood to establish the extent of the CRH-induced increase in release. Compared with unaffected individuals, depressive patients show significantly higher concentrations of cortisol and ACTH following the CRH injection. Hence the Dex/CRH test has been acknowledged as a reliable marker for a depression. Besides indicating an existing major depression, the test also helps to predict and validate the success of a given antidepressive therapy, since its results revert to normal as the major depression remits. Finally with the help of the test, predictions can also be made as to the probability of relapse [92]. Why the test is so concise is not yet fully clear. As one, rather probable explanation, a hereditary or acquired defect in the glucocorticoid receptor making it insensitive to cortisol has been discussed [93]. Given this hypothesis and since the glucocorticoid system seems to play a significant role in other mental disorders too, we should go beyond purely establishing the amount of a given hormone in the blood and have a closer look on the function of this receptor and its intracellular signal chain.

As has just been said, the glucocorticoid system seems to be a significant factor in other mental disorders, apart from depression. Compared to unaffected individuals, patients suffering from a post-traumatic stress disorder (PTSD) for example show a significantly reduced secretion of cortisol after 24 h, a lower basal concentration of cortisol, a significantly increased amount of glucocorticoid receptors on the lymphocytes and a significantly increased sensitivity to dexamethasone and its stress-hormone inhibiting effects [93]. At present it is not yet clear, but investigated, whether this unfavourable feedback is a result of the trauma, a consequence of the existence of the PTSD or whether it is already there before a trauma and hence an indicator for the probability to develop a PTSD.

Apart from affective disorders/depression and PTSD, the HPA system and its inducibility by stress (hormones) also seems to play a role in the pathophysiology of alcohol dependence. It is a normal reaction of the body’s immune system to boost the production of CRH and vasopressin in situations of social stress. With individuals dependent on alcohol the amount of both CRH and vasopressin released is significantly increased compared to unaffected individuals, whereas under normal conditions their CRH and vasopressin concentrations are significantly lower. Hence alcoholics’ reaction to social stress is much stronger than in unaffected individuals. Most probably the discrepancies in both the “normal” CRH and vasopressin concentrations and their changes under stress are innate. Individuals suffering from an alcohol dependence also show a much stronger response in the Dex/CRH test when being injected CRH. As a result, the concentration of stress hormones and hence the activation of the HPA system are much higher than in the control group. There is a third marked difference with regard to the HPA system that may play a role in alcohol dependence. In individuals with a higher risk to develop alcohol dependence, the reduction in the amounts of CRH and vasopressin produced following the consumption of alcohol is much higher than in control individuals. This has been suggested to mean that people susceptible to develop alcohol dependence experience a much bigger reduction in stress when using alcohol than individuals not showing this risk. This hypothesis is supported by the results of pharmacological tests in animals. After having been applied a CRH antagonist, rats conditioned to be alcohol-dependent showed a substantial decrease in the amount of alcohol consumed [94,95,96,97,98].

Other hormones have been suggested to play a significant role in the explanation of mental disorders. As mentioned above, in phases of hormonal rearrangements women are more likely to develop a dysphoric disorder, a gestational depression, a postpartum depression or other psychosis. The perimenopausal phase, the time following an ovarectomy or hysterectomy have been shown to increase that risk even more, while in the postmenopausal phase the risk seems to be significantly lower [50]. This has led to suggestions that estrogens seem to have a high anxiolytic and antidepressive effect, which is mediated by the serotonin system [99,100]. The fact that older men are also more likely to develop depression has also been explained by the decrease in the concentration of sexual hormones, in particular of testosterone, in their blood. Likewise, substantial evidence has been collected suggesting that a testosterone-replacing therapy is effectively anti-depressive [101].

### 4.2. Current Hormone-Based Concepts of Other Psychiatric Disorders

Male sexual hormones also seem to play a role in the pathophysiology of ADHD, namely by influencing the development of dopaminergic neuronal systems. This theory is supported by several facts. On the one hand, the prevalence of ADHD is three times higher in boys than in girls. On the other a higher exposure to testosterone before birth has been shown to go along with a higher risk to develop ADHD. Finally, androgens have been shown to have an impact on the striatum, including the nucleus caudatus and other dopaminergic structures, while all these structures are different in children with ADHD than in those without [102].

Experimental and natural field studies have strengthened evidence for suggesting that using androgens as anabolic steroids can induce hypomanic or manic phases that are often accompanied by increased aggression and use of violence [103]. In 1995 Malone *et al.* made a survey of 164 weight lifters and bodybuilders that had used androgens as anabolic steroids and found 10% of them to show or have shown hypomanic states [104]. By contrast, 10% of those who had stopped taking androgens had developed depression, which is the opposite of a manic phase. Lithium, a drug against mania, has been found to reduce the reduction of androgens in the frontal cortex and the hippocampus [105].

Likewise androgens have shown to disinhibit behavior in mice [106]. This goes along with the above-mentioned experience of people in the Neolithic period, who found that oxen were less aggressive and less disinhibited than bulls that had not been castrated, which proved unsuitable for employment for farm work.

What has also been mentioned above is the therapeutic use of sexual hormones in hypersexuality and sexually deviant behavior. It should be added here that in transsexualism it is of decisive importance to apply sexual hormones of the opposite sex. In Germany, the administration of sexual hormones in transsexuals can be started after the patient has completed 12 months of psychotherapeutic treatment. After a total of 18 months, *i.e.*, 12 months of initial psychotherapeutic treatment and six months of both hormone treatment, further psychotherapy and practical test of life in the other sex’s role, the sex change operation can be undertaken [107].

In the historical introduction, distinctive mental problems accompanying both hyper- and hypothyroidism have already been elaborated upon. At present, thyroid hormones are also used in augmenting the efficiency of antidepressive treatment—or making it possible in the first place. In practice, up to one third of patients suffering from major depression do not respond at all or only insufficiently to conventional treatment with antidepressants [108]. Several studies among euthyroid depressive patients, *i.e.*, patients with a normal functioning of the thyroid, not or only insufficiently responding to psychopharmaceutical treatment have shown the efficacy of applying triiodothyronine or levothyroxine as augmentors to make antidepressive treatment (more) effective [109]. In a meta-analysis of eight studies, Aronson *et al.* have found that twice as many patients were responsive to antidepressive treatment augmented by triiodothyronine compared to patients receiving conventional antidepressive treatment only [110]. On the other hand, Altshuler *et al.* found a faster response to antidepressive treatment with imipramine or amitriptyline when 25–50 μg triiodthyronine were applied simultaneously [111].

For the pathophysiology of eating disorders the hormones that are responsible for regulating appetite are especially relevant. The hypothalamus plays a vital part in regulating body weight. On the one hand it integrates the signals received from the periphery on both the nutritional status, the saturation level and food intake, and on the other it modulates food intake and energy consumption. Anorexiant (appetite suppressing) signals include glucose, the pancreas hormone insulin, the fatty tissue hormone leptin and the cytokine TNF-α, while ghrelin, which is produced in the stomach, stimulates appetite (orexigenic signal). In the hypothalamus now the arcuate nucleus, the paraventricular nucleus and the lateral hypothalamus are particularly important for regulating body weight. The arcuate integrates the signals received by glucose, insulin, leptin, ghrelin and other hormones as well as by energy sources and transforms them into neuronal signals. This integration in the arcuate nucleus is modulated by various neurotransmitters including histamine, serotonin, dopamine, noradrenaline, glutamate and acetylcholine. The neurons of the arcuate nucleus innervate nerve cells in the paraventricular nucleus and in the lateral hypothalamus, where the signals received from the arcuate are processed. For transmitting its signals, the arcuate nucleus has two types of neurons that act as antagonists. The one type releases neurotransmitters neuopeptide Y (NPY) and agouti-related peptide (AgRP) and stimulates appetite. The other type of neuron the melanocyte-stimulating hormone (α-MSH) and the “cocaine and amphetamine regulated transcript” (CART) and downregulates or suppresses appetite [112]. Leptin is a hormone produced by fat cells to signal saturation [43]. In patients suffering from anorexia leptin levels are very low since their bodies contain only low amounts of fatty tissue. By contrast, the levels of ghrelin are significantly higher. It is assumed that both the increased levels of leptin and the reduced levels of ghrelin are caused by fasting. On the other hand anorexia patients show low concentrations of NPY which may be why they eat too little. Another hypothesis currently being discussed is that, for genetic reasons, anorexia patients produce too much TNF-α, which has a huge appetite-suppressing effect. Due to its anorexigenic effect TNF-α is also called cachexin or cachectin. The changes in the neurotransmitters, cytokines and hormones involved in appetite regulation bring about changes in other hormonal systems such as sexual hormones, and the HPA system [113,114]. The example of eating disorders shows that biological psychiatric research must not stay focused on hormonal aspects of disease, but should increase their efforts in elaborating on the interaction of neurotransmitters, the immune and the hormone system.

The appetite regulators just mentioned also seem to be involved in alcohol dependence. So far the role of leptin and ghrelin in alcohol dependence has been investigated, yet with no clear results. In people dependent on alcohol both increased [115] and decreased [116] concentrations of leptin have been found compared to unaffected individuals. Likewise the effect of alcohol intake on leptin concentrations remains unclear. Yet it must be noted that these studies may not have differentiated enough by gender, age and body weight [117], for in studies on alcohol-dependent women only a clear connection between “craving”, *i.e.*, the strong desire to drink alcohol, and the concentration of leptin in the plasma has been found [118]. In healthy individuals, both male and female, the intake of alcohol leads to a decrease in the concentration of ghrelin [119]. In individuals dependent on alcohol both decreased and increased level of this hormone have been found in studies, yet the results suggest that the decreased levels are typical of active drinkers, whereas the increased levels seem typical of teetotal former alcoholics during rehabilitation [119]. Increased ghrelin levels also seem to be characteristic for alcoholics during detoxification [120]. Finally, tests on mice have shown that the intraventricular application of ghrelin led to more alcohol being consumed, whereas the application of a ghrelin antagonist reduced the longing for alcohol [121].

### 4.3. Current Immunological Concepts of Schizophrenia

In the topical scientific view, the immune system consists of two parts: the innate immune defense and the adaptive immune defense. The innate immune system can fight pathogens without prior contact with them. The innate immune defense is accomplished by granulocytes, monocytes, macrophages, dendritic cells or natural cytotoxic (or “killer”) cells. These cells can modulate immune reaction of the body by producing cytokines. In the central nervous system, cytokines are produced by astrocytes and cells in the microglia. Cytokines influencing the brain include interleukins (IL)-1 and IL-6 as well as tumor necrosis factor (TNF)-α. The latter, TNF-α, seems to be crucial in activating an immune response. As its name suggests, a major feature of the adaptive immune system is its adaptability or adaptation to new pathogens [122].

Cells forming the adaptive immune system include cytotoxic T cells, B lymphocytes and T helper cells (TH). The latter T helper cells can be sub-differentiated into 2 sub-types by their secretion of cytokines, the TH1 cells producing IL-2 and interferon (IFN)-γ, while TH2 release IL-4, IL-5 and IL-10. While TH1 activates cellular immune defence, TH2 induces the production of antibodies, *i.e.*, a humoral immune response [122,123]. The current psychoimmunological concepts of depression suggests an increase in the TH1 response, while in schizophrenia the TH2 response seems to be increased [124]. In patients suffering from schizophrenia a decrease in the *in vitro* production of IL-2 and lower concentrations of IFN-γ have been found which seems to suggest a decrease in TH1 response, while the TH2 response seems to be increased. Pre- or postnatal infections have been suggested responsible for the typical immunological characteristics of schizophrenic patients.

The increase in type 2 immune response (TH2 response) suggests an increase in astrocyte activation and an inhibition of microglia functioning. This suggestion is supported by higher levels of S100B astrocyte marker both in the blood and the spinal fluid found in schizophrenic patients. The activation of astrocytes leads to higher levels of kynurenic acid, since astrocytes lack an enzyme called kynurenine (3)-monooxygenase (KMO) which is needed to break down kynurenine (which in itself is a break-down product of tryptopthan, an amino acid) into quinolonic acid, making astrocytes a primary source of for kynurenic acid [125].

**Figure 4:**
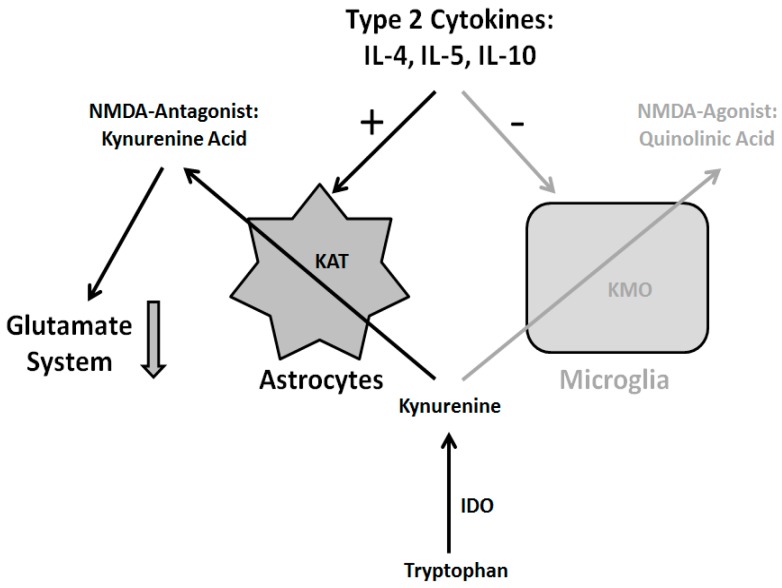
Immune hypothesis of schizophrenia: The type 2 cytokines interleukins (IL)-4, IL-5 and IL-10 activate astrocytes and inhibit microglia. Astrocytes, in turn, produce the glutamate antagonist kynurenic acid which leads to a reduction of glutamate signaling accompanied by disturbances of the dopamine system. For details see text. Abbreviations: kynurenine aminotransferase (KAT), kynurenine (3)-monooxygenase (KMO), indoleamine (2,3)-dioxygenase (IDO), *N*-methyl-d-aspartate (NMDA).

So far kynurenic acid is the only antagonist of the *N*-methyl-d-aspartate (NMDA) receptor shown to be increased in schizophrenic patients. This blocking of the NMDA receptor leads to diminished glutamate signaling. Since the functioning of the dopamine system is modulated by the glutamate system, its underfunctioning in turn leads to a malfunction of the dopamine system [124,126]. See (Figure 4) for a comprehensive overview.

The facts and hypotheses described may suggest applying immune modulators to downregulate the TH2 immune response. So far in particular downregulating cyclooxygenase-2 (Cox-2) has been proposed and indeed first tests of the Cox-2 inhibitor Celecoxib applied as an add-on to conventional antipsychotic treatment have meanwhile shown promising results [127]. Yet warnings have been raised that this new medication may only be effective in the first phases of schizophrenia, but not in patients already suffering for a longer time [126]. In fact, this observation goes well along with findings made by Wagner-Jauregg in his immuno-modulating malaria fever therapy: namely that patients suffering from “acute insanity” benefit more from this new therapy than patients “with chronic insanity” [128].

All these consideration lead to the question whether the efficiency of antipsychotic medication may be explained by a general downregulation of the TH2 response and hence a modulation of the immune system. In fact, immunomodulating effects of antipsychotic drugs have been described ever since the invention of chlorpromazine [129]. A look at frequent side effects of modern drugs like clozapine, which include fever granulocytosis, agranulocytosis, serositis and myocarditis, supports the notion of such immunomodulating effects of antipsychotic drugs [122].

Moreover, clozapine was found to stimulate production of TNF-α, since patients undergoing a therapy with clozapine show increased level of TNF-α in their plasma [130]. When this was first discovered, it was deemed a peripheral effect. Yet then it was shown that injecting clozapine into the abdominal viscera of rats led to a significant increase in TNF-α concentrations in their frontal cortex [123]. This in turn strengthens the notion that the good antipsychotic therapeutic effect of clozapine is induced by activating the TH1 cytokines and intensifying TH1 immune response.

By the time the role of autoantibodies against NMDA receptors became obvious for a specific type of encephalitis [131], few groups of researchers started to reconsider the idea of Lehmann-Facius that auto-immune antibodies could contribute to the pathogenesis of schizophrenia. For example, Margari *et al.* found significant differences in circulating autoantibodies in the hippocampus and cerebellum and in anti-nuclear autoantibodies between patients suffering from schizophrenia and healthy controls [132]. Recent important studies have examined the role of glutamate, dopamine, acetylcholine and serotonin receptor autoantibodies, and other antineuronal antibodies against synaptic proteins in the serum of patients diagnosed with schizophrenia [133] and found interesting results pointing to a possible role of these antibodies in the development in schizophrenia in at least a subgroup of patients. For example, in acutely ill patients with an initial schizophrenia diagnosis an increased prevalence of NMDA receptor antibodies could be shown [134]. There are further hints towards the involvement of the cytokine system in the development of schizophrenia: Offspring of women who experience infection while pregnant have an increased risk for schizophrenic disorders [135] which might be mediated by changes in the production of IL-6 which then lead to critical behavioral and transcriptional changes in the offspring [136,137]. Those changes in the cytokine system during fetal development may also be caused by maternal microbiota alterations [138]. Not only infections of the mother, but also earlier infections of the patient (e.g., Borna disease virus infection [139] and Toxoplasma gondii infection [140,141]) may be a predisposing factor for schizophrenia.

Yet these findings are not uncontroversial, for several studies among untreated patients suffering from schizophrenia have found increased concentrations of inflammatory cytokines such as IFN-γ and TNF-α, which suggest an intensified TH1 response [142]. Moreover, not all schizophrenic patients showed elevated autoantibodies in studies mentioned in the paragraph above. Also the concept described above is outdated in so far, as meanwhile TH-17 cells and Tregs have been discovered. Up until now no newer psychoimmunological hypothesis has been suggested that would cover and integrate all these new immunological findings.

What has been suggested though is that a major shortcoming and obstacle for a more profound role of psychiatric immunology are the huge insufficiencies of diagnosing psychiatric disorders using the diagnostic systems international classification of diseases (ICD)-10 of the World Health Organization (WHO) and Diagnostic and Statistical Manual of Mental Disorders (DSM)-5 of the American Psychiatric Organisation (APA). It was the forefather of psychiatric immunology, Wagner-Jauregg, who found that patients suffering from “acute insanity” are more likely to benefit from feverish illnesses and hence from his malaria fever therapy than patients suffering from “chronic insanity”. In today’s classification systems, however, both kinds of patients would be given the same diagnosis “schizophrenia”, even though their immunological dispositions differ substantially. Hence there is a big hope that in the future it will no longer be necessary to classify and diagnose by lists of symptoms only, but that pathogenetic findings will enhance diagnostics and deliver clear and unambiguous criteria.

### 4.4. Current Immunological Concepts of Deprezssion

Several findings suggest a clear connection between the cytokine system and depression. The therapeutic application of interferons in cancer and hepatitis C patients has revealed depressive symptoms such as tiredness, loss of appetite and cognitive losses as major side effects. In animal experiments, the application of pro-inflammatory cytokines has induced so-called “sickness behavior”, which is usually regarded an equivalent of depressive symptoms in human beings [143]. Likewise an intravenous application of endotoxin in unaffected individuals has stimulated the production of cytokines and led to affective and amnestic symptoms [144].

In a study conducted by the Weihenstephan Centre of Sciences at Munich Technical University and of the Max Planck Institute for Psychiatry in Munich, the concentrations in plasma of both TNF-α and its soluble receptors p55 and p75 were analyzed in 523 individuals that had never had a depression, in 35 individuals that had suffered from a depression before but not at the time of the study and in 70 in-patients suffering from acute depression, but without any inflammatory diseases. The lowest concentrations of both TNF-α and its soluble receptors had been found in those individuals that had never suffered from a depressive episode before. In individuals who had suffered from a depression at some point before the study, those concentrations were slightly released. By contrast the 70 patients suffering from acute depression at the time of the study showed the highest concentrations. This has led to the conclusion that TNF-α is involved in the pathophysiology of depression [145]. The finding of elevated TNF-α levels in depressed patients could recently be replicated in another cross-sectional study [146].

**Figure 5:**
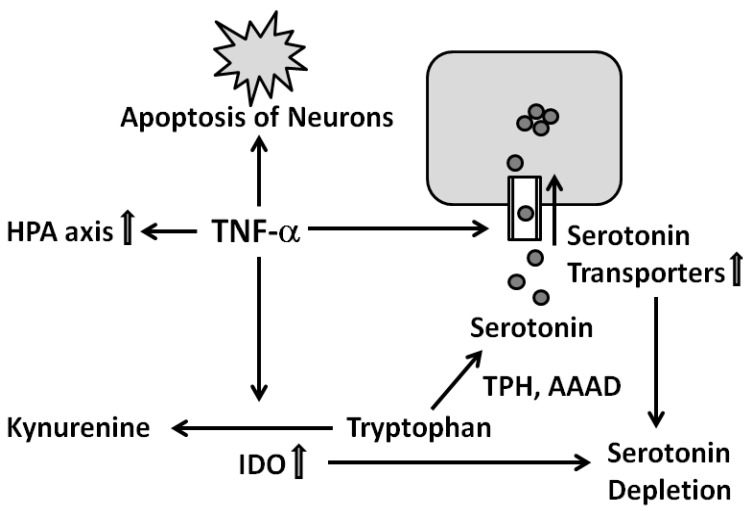
Mechanisms through which TNF-α could contribute to the emergence of a depression: activation of the hypothalamus-pituitary-adrenal (HPA) axis, induction of apoptosis, stimulation of the serotonin reuptake and induction of the IDO. For details see text. Abbreviations: tryptophan hydroxylase (TPH), aromatic l-amino acid decarboxylase (AAAD), indoleamine (2,3-)dioxygenase (IDO), tumor necrosis factor-α (TNF-α).

There are several potential ways through which TNF-α can contribute to the emergence of a depression. On the one hand, TNF-α activates the hypothalamus-pituitary-adrenal (HPA) axis, with an activated HPA axis being one of the most consistent neurobiological features shown by depressive patients. On the other hand, TNF-α could induce apoptosis of neurons. Thirdly, TNF-α has shown to increase the re-absorption of serotonin, so that the availability of serotonin in the brain is reduced. Most probably the higher reuptake of serotonin is induced by an increase in the production of more or by activating the existing serotonin transporters. In fact, the opposite, namely the inhibition of serotonin reuptake in order to increase its availability is the working principle behind several antidepressants. Finally TNF-α has also been shown to activate indoleamine (2,3)-dioxygenase (IDO), which breaks down tryptophane, which is needed to synthesize serotonin, to kynurenine, so that the production of serotonin is reduced, which once again reduces its availability [147]. These mechanisms are depicted in Figure 5. It should be noted that meanwhile other pro-inflammatory cytokines, apart from TNF-α, have been shown to be increased in patients with affective disorders. Examples include IL-1 and IL-6 [147]. The concept of cytokines being crucial in the pathophysiology of depression is also referred to as the “cytokine hypothesis of depression”.

Meanwhile TNF-α-antagonists have been developed and approved for treating a variety of inflammatory diseases including psoriasis. Since TNF-α-antagonists can intercept TNF-α in the plasma, they are also assumed to have antidepressive effects. And in fact these antidepressive effects have also been shown–in patients with psoriasis [148]. It is a common fact that patients with psoriasis often suffer from depressive symptoms. In a clinical trial 618 patients suffering from psoriasis were also examined for depressive symptoms and in fact 30% also showed a clinically relevant depression. These 30% were applied 50 mg etanercept, a TNF-α blocker, or a placebo, for 12 weeks. When reassessed for their depressive symptoms using the Hamilton Depression Scale and the Beck Depression Inventory, significantly more patients of the group receiving the TNF-α blocker showed a reduction in their depressive symptoms than in the placebo group. It should be noted that for their original—and primarily tested—purpose, namely treating psoriasis, the TNF-α blocker did not come up to the expectations set. Hence the improvement in depressive symptoms cannot be attributed to an alleviation or disappearance of the dermatological symptoms. This clinical trial has raised new hopes for depressive patients, at least such who also show inflammatory diseases, benefitting from treatment with TNF-α blockers [148]. For the moment though etanacerpt has not been approved for application in depressive patients. However, it has been shown to exert antidepressant-like effects in rats [149], and a first attempt has been made to apply etanercept in patients with difficult-to-treat depression [150], but without significant therapeutic effect.

Another inhibitor of pro-inflammatory cytokines, namely the COX-2 inhibitor celecoxib, has been tested in depressive patients as an add-on therapy together with antidepressant reboxetine. After six weeks of treatment, both groups of patients, *i.e.*, those treated with reboxetine and a placebo and those treated with reboxetine and celoxib, showed improvements according to the Hamilton Depression Scale, yet the improvement was significantly higher in patients receiving celoxib as an add-on [151].

Meanwhile a number of *in vitro* studies have confirmed that antidepressants inhibit or suppress the production of pro-inflammatory cytokines. Studies include such in cultivated white blood cells and such in so-called whole blood assay. In these tests, the production of cytokines had been stimulated by lipopolysaccharides (LPS), phytohaemagglutinin (PHA) or concanavalin A (ConA) to which tricyclic antidepressants (TCA), norepinephrine/noradrenaline reuptake inhibitors, serotonin reuptake inhibitors or lithium were added [152,153]. In most of these tests, the production of pro-inflammatory cytokines was suppressed by addition of the psychopharmacological agents [152]. This has led to the conclusion that antidepressants have an anti-inflammatory effect. However, there are few recent *in vitro* investigations that found that at least some antidepressants even increase the production of pro-inflammatory cytokines [154,155]. Therefore, on should abstain from too far reaching and generalizing conclusions that all antidepressants would consistently reduce the production of pro-inflammatory cytokines.

Some years ago, as mentioned above, Tregs were identified as a new sub-population of T cells and since then several studies have revealed details about their function and role. Regulatory T cells (Tregs) play a crucial role in controlling—or better suppressing—the body’s immune response. Suppressing the body’s immune response is essential to prevent the body’s own cells from being attacked. Tregs express surface molecules, also called clusters of differentiation, CD4 and, even more, CD25, which is why Tregs are sometimes also referred to as CD4+CD25^hi^. With the help of impulse cytophotometry (see above) Tregs can be identified in the blood using specific antibodies for these CDs. The Tregs’ immuno-modulating capabilities are arranged for by molecules in the membranes, but also by the name-giving regulation or modulation of cytokine production [156].

We established the concentrations of Tregs and IL-1 in the plasma and analyzed how the production of cytokines IL-1 and IL-6 could be stimulated by lipopolysaccharides (LPS) [157]. The study included 16 patients suffering from depression who were tested for the concentrations of Tregs and IL-1 in their plasma at the beginning of the study, before treatment, and after six weeks of treatment. Furthermore, the severity of their depression was established at those two points with the help of the Hamilton Depression Scale (HAMD-21). The Tregs concentration was established with the help of specific antibodies against CD4 and CD25 in an impulse cytophotometer. Over the course of six weeks the average decrease in depressive symptoms was from 16 to 7 on HAMD-21. Also all patients showed a decrease in IL-1 concentration down to below the minimum detectable level over those six weeks. At the same time the concentration of Tregs in their plasma increased significantly. It has been suggested that the decrease in IL-1 was brought about by the regulatory T cells whose concentration in turn was increased by the antidepressant applied–and that hence antidepressants inhibit the production of pro-inflammatory cytokines [157].

## 5. Perspectives of Immunoendocrine Concepts of Psychiatric Disorders and Their Therapy

The historical background covered above, brings us to the present and immediate future of the field of immunoendocrinology in psychiatry. What are some of the discernible trends and challenges?

Regarding novel therapies deriving from endocrine disease concepts, both corticotropin-releasing hormone (CRH) antagonists as well as glucocorticoid receptor (GR) antagonists are being tested clinically for their antidepressive effects [158]. This raises hopes that in the future hormones may not only play a role in diagnosing, but also treating depressive patients. Apart from the hormones mentioned and discussed above, both male and female sexual hormones, and the vasopressin, leptin and neurokinin systems [101,158,159] seem to be dysregulated in depressive patients, so that accordingly neurokinin and vasopressin antagonists are also being tested as antidepressants. Regarding immunological therapies, modulators of the immune system [127,151] as well as cytokine antagonists [149] have revealed promising results regarding the therapy of depression as well as schizophrenia, but at the moment, none of these novel agents are approved in these indications.

In the future it will not suffice to know one particular cytokine or hormone level, rather it will be crucial to establish the main markers for the different hormonal or cytokine networks. These markers depend on both hereditary determinants and on factors acquired during the lifetime. Given this complexity, purely hormonal or immunological concepts of therapy or illness will not be adequate. Rather, besides biological factors, a systematic approach to mental illness must include pathophysiological, behavioral, depth-psychological and developmental psychological perspectives of mental illness.

To date, progress in psychiatric endocrinology and immunology has been largely predicated on developments in technology. Hence it can be assumed that the steady progress including newly introduced high-throughput screening together with advances made in both genomic and proteomic methods will lead to more biomarkers being identified and verified, improved lab-diagnostic methods and to new drug targets being discovered. One big challenge will be to establish high-quality sets of data on test individuals and patients in research and psychiatric practice that are readily available, reliable, not too expensive and still sensitive–and, last but not least, achievable.

It is already clear that the profusion of methods to analyse and establish the genomic, epigenetic, proteomic, hormonal and immunological parameters and for neurophysiological testing together with the different methods of structural and functional imaging will be too much for one individual psychiatrist or neuroscientist to master. Hence it is essential to consider analysis techniques that preprocess and merge the results of individual tests to produce composite results. It is likely that the bigger challenge for developing hormonal and immunological concepts of therapy and illness will not lie primarily in finding new hormones, but rather in managing the vast amounts of data that are already available.

Apart from the hormones, cytokines and immunological cells discussed above, a variety of other parameters have been discovered that effect the central nervous system. However, these molecules, their receptors and intracellular signal chains are beyond the scope of this survey, even though they have to potential to offer psychoendocrinological research new perspectives including new drug targets and hence new therapies.
